# Comparison of Dried Blood Spots and Venous Blood for the Detection of SARS-CoV-2 Antibodies in a Population of Nursing Home Residents

**DOI:** 10.1128/Spectrum.00178-21

**Published:** 2021-09-22

**Authors:** Eline Meyers, Stefan Heytens, Asangwing Formukong, Hanne Vercruysse, An De Sutter, Tom Geens, Kenneth Hofkens, Heidi Janssens, Eveline Nys, Elizaveta Padalko, Ellen Deschepper, Piet Cools

**Affiliations:** a Department of Diagnostic Sciences, Faculty of Medicine and Health Sciences, Ghent University grid.410566.0grid.5342.0grid.410566.0 , Ghent, Belgium; b Department of Public Health and Primary Care, Faculty of Medicine and Health Sciences, Ghent University grid.410566.0grid.5342.0grid.410566.0 , Ghent, Belgium; c Research and Analytics, Liantis Occupational Health Services, Bruges, Belgium; d Laboratory of Medical Microbiology, Ghent University Hospital, Ghent, Belgium; e Biostatistics Unit, Faculty of Medicine and Health Sciences, Ghent University grid.410566.0grid.5342.0grid.410566.0 , Ghent, Belgium; Quest Dianostics Nicols Institute

**Keywords:** elderly, nursing homes, SARS-CoV-2, COVID-19, antibodies, immunoglobins, IgG, serology, dried blood spots, enzyme-linked immunosorbent assay, ELISA, serosurveillance

## Abstract

In the current severe acute respiratory syndrome coronavirus 2 (SARS-CoV-2) pandemic, testing for SARS-CoV-2-specific antibodies is paramount for monitoring immune responses in postauthorization vaccination and seroepidemiological studies. However, large-scale and iterative serological testing by venipuncture in older persons can be challenging. Capillary blood sampling using a finger prick and collection on protein saver cards, i.e., dried blood spots (DBSs), has already proven to be a promising alternative. However, elderly persons have reduced cutaneous microvasculature, which may affect DBS-based antibody testing. Therefore, we aimed to evaluate the performance of DBS tests for the detection of SARS-CoV-2 antibodies among nursing homes residents. We collected paired venous blood and DBS samples on two types of protein saver cards (Whatman and EUROIMMUN) from nursing home residents, as well as from staff members as a reference population. Venous blood samples were analyzed for the presence of SARS-CoV-2 IgG antibodies using the Abbott chemiluminescent microparticle immunoassay (CMIA). DBS samples were analyzed by the EUROIMMUN enzyme-linked immunosorbent assay (ELISA) for SARS-CoV-2 IgG antibodies. We performed a statistical assessment to optimize the ELISA cutoff value for the DBS testing using Youden's J index. A total of 273 paired DBS-serum samples were analyzed, of which 129 were positive, as assessed by the reference test. The sensitivities and specificities of DBS testing ranged from 95.0% to 97.1% and from 97.1% to 98.8%, respectively, depending on the population (residents or staff members) and the DBS card type. Therefore, we found that DBS sampling is a valid alternative to venipuncture for the detection of SARS-CoV-2 antibodies among elderly subjects.

**IMPORTANCE** Since the implementation of newly developed SARS-CoV-2 vaccines in the general population, serological tests are of increasing importance. Because DBS samples can be obtained with a finger prick and can be shipped and stored at room temperature, they are optimal for use in large-scale SARS-CoV-2 serosurveillance or postauthorization vaccination studies, even in an elderly study population.

## INTRODUCTION

In the current severe acute respiratory syndrome coronavirus 2 (SARS-CoV-2) pandemic, large-scale serological antibody studies are paramount to assess the true SARS-CoV-2 infection rate. Indeed, statistics on PCR-confirmed SARS-CoV-2 cases are far from ideal to estimate the true proportion of the population that has experienced a SARS-CoV-2 infection (1), as mildly affected or asymptomatic individuals are often not tested and PCR-based testing yields only an epidemiological snapshot. Furthermore, the recent implementation of newly developed SARS-CoV-2 vaccines creates an urgent need for population-based serological studies to monitor antibody responses postauthorization. Protocols for SARS-CoV-2 antibody assays using serum/plasma obtained by venipuncture are well established in a clinical setting. However, venipuncture is invasive and can cause serious discomfort. Especially for elderly subjects, venipuncture poses large challenges due to dehydration, loss of vein patency, and low blood pressure. Elderly subjects may also suffer from arthritis, injury, or stroke, impeding hyperextension of the arms to survey for available veins. The use of venipuncture is further limited for wider application in nonclinical settings due to the costs (e.g., for phlebotomists) and logistical constraints associated with collecting, processing, and transporting venous blood.

Capillary blood collection on protein saver cards after finger pricking using a lancet, the so-called dried blood spot (DBS), is a most valuable alternative because it is minimally invasive, can be performed at low cost, and has the potential for self-sampling and samples are easy to ship and store. DBSs are increasingly being applied as a minimally invasive alternative for infectious serological testing, especially in community- and population-based epidemiological studies, including for SARS-CoV-2 (2). However, the use of DBSs for detection of SARS-CoV-2 antibodies in elderly subjects has not yet been validated. Aging is known to modify the cutaneous microvasculature and the structures of blood vessels, even to the level of the capillary basement membrane (3). Here, we aimed to validate the use of DBS sampling for the detection of SARS-CoV-2 IgG antibodies in a population of residents and staff members from nursing homes (NHs).

## RESULTS

**FIG 1:**
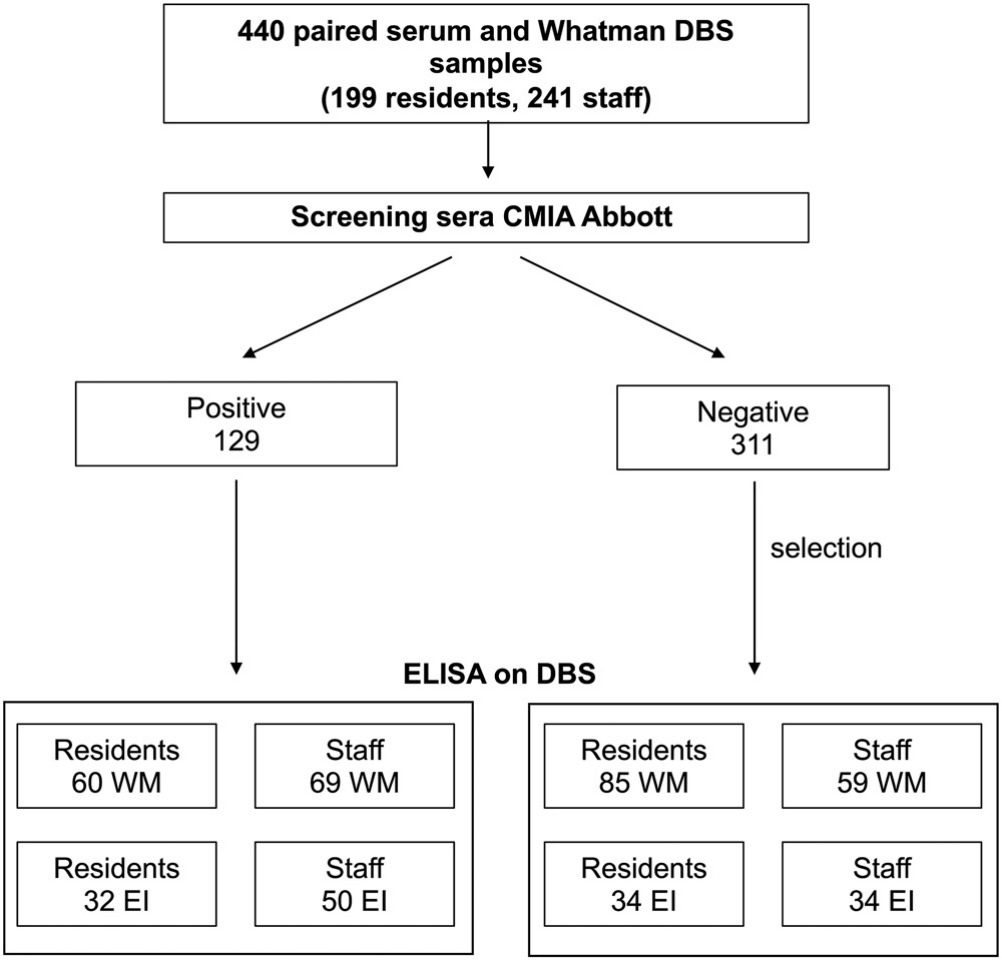
Schematic diagram of the collection, screening, and analysis of serum and DBS samples. EI, EUROIMMUN; WM, Whatman.

In four NHs, a total of 440 paired venous blood-Whatman DBS samples were obtained, of which 199 were from residents and 241 from staff members (Fig. 1). The sera from these 440 venous blood samples were screened by means of the Abbott IgG chemiluminescent microparticle immunoassay (CMIA) reference assay. Of these samples, 129 samples (from 60 residents and 69 staff members) were found to be positive. The paired Whatman DBS samples for these positive sera, together with 144 paired Whatman DBS samples for negative sera, were analyzed by means of enzyme-linked immunosorbent assay (ELISA). The selection of paired DBS samples from negative sera included 85 paired samples from residents and 59 from staff members. The mean ages of the residents and staff members for which the paired serum-Whatman DBS samples were analyzed were 87.8 years (range, 67 to 100 years) and 42.8 years (range, 19 to 65 years), respectively. A total of 80.0% and 93.8% of residents and staff members, respectively, were female. Additionally, a subset of 150 EUROIMMUN DBS samples were analyzed, of which 82 samples (from 32 residents and 50 staff members) were found to be positive by the reference test (Fig. 1).

The numbers of true and false positive and negative results for the Whatman DBS and EUROIMMUN DBS samples, in comparison with the reference test, for all participants and categorized according to residents versus staff, are shown in Table 1. One of the two false-positive Whatman DBS test results, with an optical density (OD) value of 4.39, was obtained from a participant who tested positive (by reverse transcription [RT]-PCR) for SARS-CoV-2 in March 2020, making it unlikely that this DBS seropositivity was a false-positive result; the reference test result likely was a false-negative result. No RT-PCR data were available for the other discordant samples. The sensitivity and specificity of both the Whatman DBS and EUROIMMUN DBS tests, compared to the Abbott CMIA as a reference test, are shown in Table 2.

**TABLE 1:** Numbers of SARS-CoV-2 IgG true/false-positive and true/false-negative Whatman DBS and EUROIMMUN DBS test results, in comparison with Abbott CMIA reference test results

Group and test result	No. with reference test result of^*a*^:	
	Positive	Negative
Whatman DBS		
Total (*n* = 273)		
Positive	124	2
Negative	5	142
Residents (*n* = 128)		
Positive	57	1
Negative	3	84
Staff members (*n* = 145)		
Positive	67	1
Negative	2	58
EUROIMMUN DBS		
Total (*n* = 150)		
Positive	78	2
Negative	4	66
Residents (*n* = 66)		
Positive	31	1
Negative	1	33
Staff members (*n* = 84)		
Positive	47	1
Negative	3	33

a The reference test was the CMIA with venous blood samples.

**TABLE 2:** Sensitivity and specificity of the Whatman and EUROIMMUN DBS ELISAs, in comparison with the Abbott CMIA as the reference test

DBS and group	Sensitivity (95% CI) (%)	Specificity (95% CI) (%)
Whatman DBS		
Total	96.1 (91.2–98.7)	98.6 (95.1–99.8)
Residents	95.0 (86.3–98.6)	98.8 (93.6–99.9)
Staff members	97.1 (90.0–99.5)	98.3 (91.0–99.1)
EUROIMMUN DBS		
Total	95.1 (88.1–98.1)	97.1 (89.9–99.5)
Residents	96.7 (84.3–99.8)	97.1 (85.1–99.9)
Staff members	94.0 (83.8–98.4)	97.1 (85.1–99.9)

**FIG 2:**
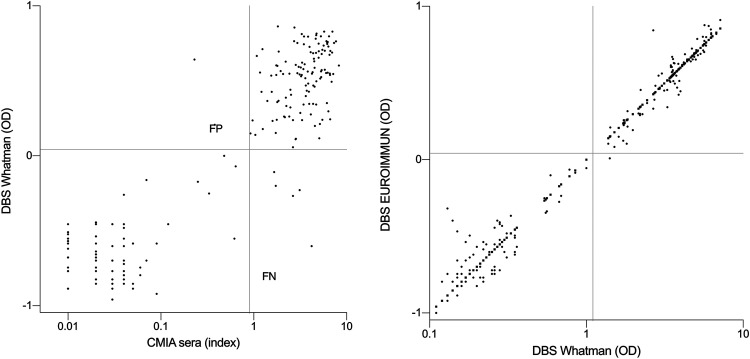
Scatterplots of paired serum sample and Whatman DBS sample (left) and Whatman DBS sample and EUROIMMUN DBS sample (right) OD/index values. Axes represent the log_10_ OD and index values. FN, false negative; FP, false positive. The gray horizontal and vertical lines represent cutoff lines defining positive samples.

The scatterplot of the index values for the reference test (Abbott CMIA) and the OD values of the Whatman DBS ELISA is shown in Fig. 2. The Pearson correlation coefficient for the reference test and the Whatman DBSs was 0.80 (95% confidence interval [CI], 0.75 to 0.84) and significant (*P* < 0.0001). The Pearson correlation coefficient for the reference test and the EUROIMMUN DBSs was 0.78 (95% CI, 0.73 to 0.82) and significant (*P* < 0.0001) (the scatterplot was similar to that for the reference test versus the Whatman DBSs [data not shown]). A scatterplot showing the correlation between the OD values from the 150 paired Whatman and EUROIMMUN DBSs is depicted in Fig. 2. The Pearson correlation coefficient was found to be 0.970 (95% CI, 0.959 to 0.978) and significant (*P* < 0.0001). For one sample pair, the Whatman DBS showed a positive result (OD of 1.41) but the EUROIMMUN DBS did not (OD of 1.02). However, the latter result is classified as borderline according to the manufacturer’s guidelines; this category was not considered in the current analysis, and this result was classified as negative.

**FIG 3:**
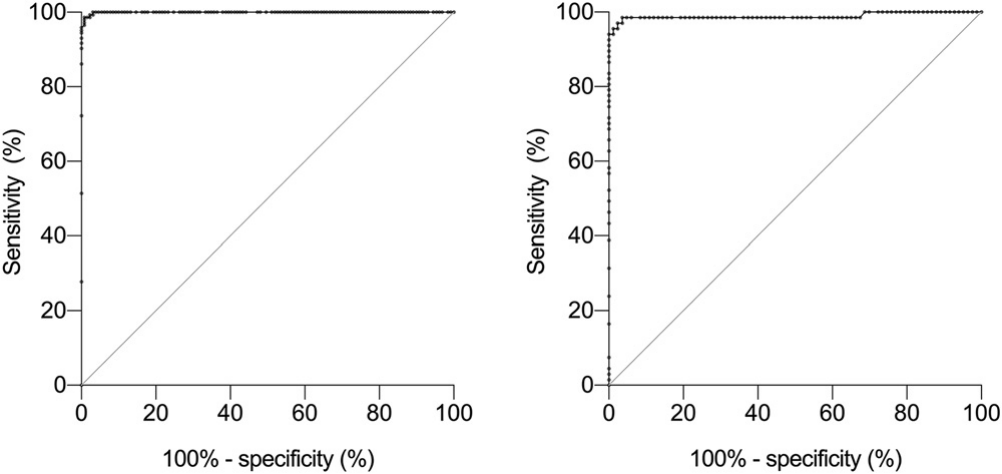
ROC curves for evaluation of the Whatman DBS ELISA (left) and the EUROIMMUN DBS ELISA (right), compared to the reference assay. The AUC is 0.999 for both Whatman and EUROIMMUN DBSs (95% CI, 0.999 to 1.000) (*P* < 0.0001).

To verify whether the ELISA Whatman DBS and EUROIMMUN DBS cutoff values could be optimized, we performed a receiver operating characteristic (ROC) analysis. The ROC curves are shown in Fig. 3. The area under the curve (AUC) was 0.999 for both Whatman and EUROIMMUN DBSs (95% CI, 0.999 to 1.000) (*P* < 0.0001). The optimal cutoff point for the Whatman DBSs was found to be 1.14, which is almost exactly the value of 1.1 proposed by the manufacturer of the ELISA, and did not improve sensitivity or specificity. The optimal cutoff point for the EUROIMMUN DBSs was found to be 1.02, and applying this cutoff value improved both sensitivity (from 95.1% to 96.4%) and specificity (from 97.1% to 98.5%).

## DISCUSSION

In the present study, we evaluated the use of DBSs for detection of SARS-CoV-2 antibodies in residents and staff members from NHs. To the best of our knowledge, we report the largest clinical DBS validation study, comparing two different DBS samples (Whatman and EUROIMMUN) in a key target population in the current SARS-CoV-2 pandemic, namely, elderly persons residing in NHs.

Overall, we demonstrated high sensitivity (range, 95.0 to 97.1%) and specificity (range, 97.1 to 98.8%) for DBS samples, compared to serum samples. Adjustment of the manufacturer-recommended cutoff value from 1.1 to 1.02 resulted in slight improvements in sensitivity and specificity for the EUROIMMUN DBS test only. We found no significant differences in sensitivity or specificity of the DBS tests between residents and staff members, for both Whatman and EUROIMMUN DBS, as demonstrated by the largely overlapping CIs. Furthermore, Whatman and EUROIMMUN protein saver cards yielded results that were in nearly perfect agreement.

Several other studies in different populations, such as health care workers, key workers, athletes, and children, have evaluated DBS sampling for SAR-CoV-2 antibody testing (4–12). In those studies, similar test characteristics were reported, with sensitivity values ranging from 89% up to 100% and specificity values of 100% (5, 8, 9), or nearly perfect agreement in SARS-CoV-2 antibody detection between DBS samples and paired venous blood samples was reported (4, 7, 10–12). However, some studies were limited by the lack of serum serological testing as a reference test (5, 6) or a rather limited sample size (i.e., <50 positive results, as assessed by the reference test) (4, 5, 9, 10).

Our findings show excellent diagnostic performance of DBS testing for both elderly patients and staff members from NHs. This supports the use of DBSs in large-scale SARS-CoV-2 serosurveillance studies as a valuable alternative to venipuncture, especially for elderly subjects, for whom venous blood can be challenging to obtain. In comparison to rapid serological tests, which can also be implemented in large-scale SARS-CoV-2 seroepidemiological studies, DBS tests offer higher sensitivity and specificity and the possibility of (semi-)quantitatively assessing the antibody response (13). Moreover, DBS tests have the advantage of collection and storage of the samples for multiple analyses. In this way, different assays can be performed with the same sample, such as antibody assays directed against different antigens. However, DBS tests have advantages similar to those of rapid tests, as they eliminate the need for health care professionals during sampling and the need for complex shipment and storage.

Especially since the implementation of newly developed SARS-CoV-2 vaccines, serological studies are of increasing importance to fill existing research gaps. To date, the stability and duration of the antibody responses upon vaccination are unknown; however, it is important to determine whether annual boosting is needed. Furthermore, a correct antibody response cutoff value that refers to clinical immunity against SARS-CoV-2 is lacking. Additionally, it is of crucial importance to assess the effectiveness of the vaccines in the general population, which can differ from values measured in standardized clinical trials. In this context, the noninvasiveness of DBS testing, the minimal logistic constraints, the excellent test characteristics, and the possibility of quantitative assessment bring added value for conducting serosurveillance studies.

Our study was limited by the use of the CMIA with venous blood samples, which assessed nucleocapsid antibodies and not spike antibodies, which were detected by means of ELISA with DBS samples. Conflicting results are available concerning the degree of persistence between antibodies directed against the nucleocapsid and spike antigens; however, it has been suggested that levels of IgG antibodies directed against the nucleocapsid antigen wane more rapidly postinfection than those directed against the spike antigen (14). Nevertheless, this should not impact the sensitivity analysis in the current results, as nucleocapsid antibodies were detected as the reference.

## MATERIALS AND METHODS

### Ethical considerations.

The current study was approved by the Ethical Committee of the Ghent University Hospital (reference number BC-07665) and was conducted according to the principles outlined in the Declaration of Helsinki. Each participant signed an informed consent form after being informed about the goal of the study and the sampling procedures. A confidential counselor, such as a nurse, signed for participants who were incapable of signing the consent form, such as residents with dementia, when consent was given by their legal representatives.

### Study population.

In August 2020, before the start of the vaccination campaign, we contacted the management of four NHs within our network (Amphora, Wingene; Sint-Rafaël, Liedekerke; Sint-Jozef, Assenede; and Sint-Jozef, Wetteren; all in Flanders, Belgium) and explained the goal of the study. We recruited NHs that had experienced a SARS-CoV-2 outbreak in the period of March to July 2020, in order to increase the likelihood of obtaining seropositive samples, thus minimizing the number of screened participants needed to obtain our calculated sample size. The management informed the families of residents and recruited a total of 199 residents and 241 staff members. All interested staff members and residents, except for residents with severe dementia (exclusion criterion), were eligible to participate in the study.

### Sample collection.

We obtained approximately 5 ml of venous blood from each participant, in serum tubes, by venipuncture using a 23-gauge scalp vein set. Capillary blood was collected onto Whatman protein saver cards (GE Healthcare Sciences, Cardiff, UK) after briefly puncturing the top edge of the distal phalanx of the middle or ring finger using 18-gauge safety lancets (Sarstedt, Numbrecht, Germany). EUROIMMUN protein saver cards (PerkinElmer Health Sciences Inc., Lübeck, Germany) were marketed during sample collection and were evaluated together with the Whatman protein saver cards for a subset of participants. For each protein saver card, a preprinted circle was filled until saturated (i.e., blood was visible on the back of the card). In order to avoid sampling bias, for one-half of the participants (i.e., those with an even participant identification number), first a circle was filled on the Whatman protein saver card and then a circle was filled on the EUROIMMUN protein saver card; for the other one-half of the participants (those with an odd number), the opposite was done. Typically, an average of four blood drops were needed to saturate one circle. All blood collections were done under aseptic conditions. DBS samples were obtained by allowing the capillary blood to air dry on the protein saver cards for 1 h at room temperature.

Serum tubes were transported to the Laboratory of Clinical Microbiology of the Ghent University Hospital (Ghent, Belgium) within 6 h after sample collection. Upon arrival, serum tubes were centrifuged at 3,000 × *g* for 8 min, and the tubes were stored at 4°C. The following day, serum was aliquoted into new serum vials and analyzed by means of the SARS-CoV-2 IgG Architect immunoassay (Abbott Laboratories). DBS samples selected for analysis (see below) were analyzed a maximum of 2 days after collection.

### SARS-CoV-2 IgG detection in serum samples by means of CMIA.

All serum samples were analyzed for antinucleocapsid SARS-CoV-2 IgG serological results by using the Architect i2000sr Plus system (Abbott). This system allows high-throughput screening of the sera, providing real-time information on the number of positive samples. This way, we could rapidly validate DBS tests for our ongoing and future studies in NHs. The Architect i2000sr Plus system uses the CMIA technique to detect antibodies. After thawing the sera and vortex-mixing them briefly, the Architect system analyzes the samples automatically using a SARS-CoV-2 assay, a specific calibrator kit, and a specific control kit. We used an in-house-validated cutoff index of 0.9 to classify sera as positive (≥0.9) or negative (<0.9) for SARS-CoV-2 IgG antibodies.

### SARS-CoV-2 IgG detection in DBS samples by means of ELISA.

The DBS samples were analyzed for the presence of antispike (S1 antigen) SARS-CoV-2 IgG antibodies by means of ELISA (EUROIMMUN; PerkinElmer Health Sciences Inc.) and not the Abbott CMIA because the latter assay requires a volume of 150 μl, which is more than the volume of capillary blood absorbed on one circle of the DBS card. One circle (6-mm diameter) was cut out from each DBS card using a puncher and was placed in a well of a sterile 96-well U-shaped microtiter plate. To avoid cross-contamination, the puncher was cleaned between punches using a 70% alcohol solution and a cotton swab. A total volume of 250 μl preheated (1 h at 37°C) sample buffer was added to each sample well of the 96-well microtiter plate, and the plate was incubated at 37°C for 1 h. After gentle mixing of the eluate by means of up-and-down pipetting, a total of 100 μl of this eluate was used for ELISA, according to the manufacturer’s instructions. The ELISA was run on the automated Behring Elisa Processor III (Siemens AG, Munich, Germany). DBS samples were classified according to their antibody index OD value (450 nm) as negative (<1.1) or positive (≥1.1), as recommended by the manufacturer. The borderline category was not considered.

### Sample size and analysis.

The sample size was calculated using the methodology described by Buderer, focusing on the sensitivity (15). Here, we hypothesized that the sensitivity of DBS testing would be lower than that of serum testing due to antibodies being affected by or remaining captured in the protein saver cards. Using an anticipated sensitivity of 85%, an α level of 0.05, and a precision parameter (ε) of 0.10, we needed a minimum of 49 positive serum samples, which were collected for both the residents and the staff members.

The sensitivity and specificity of the DBS tests were calculated using the Abbott CMIA with sera from venous blood samples as a reference test. The 95% CIs were calculated using the Wilson-Brown method (16).

To determine the optimal cutoff value for the Whatman and EUROIMMUN DBS samples, we calculated Youden’s J index. The accuracy was calculated using the AUC of the ROC curves. All analyses were performed using the statistical software GraphPad Prism 6 (GraphPad Software Inc., San Diego, CA, USA).
